# Lifestyle factors and site-specific risk of hip fracture in community dwelling older women – a 13-year prospective population-based cohort study

**DOI:** 10.1186/1471-2474-13-173

**Published:** 2012-09-14

**Authors:** Mikko Määttä, Erkki Terho, Heikki Jokinen, Pasi Pulkkinen, Juha Korpelainen, Jorma Heikkinen, Sirkka Keinänen-Kiukaanniemi, Timo Jämsä, Raija Korpelainen

**Affiliations:** 1 Department of Medical Technology, University of Oulu, PO Box 5000, FI-90014, Oulu, Finland; 2 Infotech Oulu, University of Oulu, PO Box 4500, FI-90014, Oulu, Finland; 3 Department of Sports and Exercise Medicine, Oulu Deaconess Institute, Kajaaninkatu 17, FI-90100, Oulu, Finland; 4 Oulu University Hospital, Kajaanintie 50, FI-90029, Oulu, Finland; 5 Osteoporosis Clinic, Oulu Deaconess Institute, Albertinkatu 16, FI-90100, Oulu, Finland; 6 Institute of Health Sciences, University of Oulu, PO Box 5000, FI-90014, Oulu, Finland; 7 Unit of General Practice, Oulu University Hospital, Kajaanintie 50, FI-90029, Oulu, Finland; 8 Department of Diagnostic Radiology, Oulu University Hospital, Kajaanintie 50, FI-90029, Oulu, Finland

**Keywords:** Fracture type, Cervical fracture, Trochanteric fracture, Mobility, Physical activity, Risk factors

## Abstract

**Background:**

Several risk factors are associated to hip fractures. It seems that different hip fracture types have different etiologies. In this study, we evaluated the lifestyle-related risk factors for cervical and trochanteric hip fractures in older women over a 13-year follow-up period.

**Methods:**

The study design was a prospective, population-based study consisting of 1681 women (mean age 72 years). Seventy-three percent (n = 1222) participated in the baseline measurements, including medical history, leisure-time physical activity, smoking, and nutrition, along with body anthropometrics and functional mobility. Cox regression was used to identify the independent predictors of cervical and trochanteric hip fractures.

**Results:**

During the follow-up, 49 cervical and 31 trochanteric fractures were recorded. The women with hip fractures were older, taller, and thinner than the women with no fractures (p < 0.05). Low functional mobility was an independent predictor of both cervical and trochanteric fractures (HR = 3.4, 95% CI 1.8-6.6, and HR = 5.3, 95% CI 2.5-11.4, respectively). Low baseline physical activity was associated with an increased risk of hip fracture, especially in the cervical region (HR = 2.5, 95% CI 1.3-4.9). A decrease in cervical fracture risk (p = 0.002) was observed with physically active individuals compared to their less active peers (categories: very low or low, moderate, and high). Moderate coffee consumption and hypertension decreased the risk of cervical fractures (HR = 0.4, 95% CI 0.2-0.8, for both), while smoking was a predisposing factor for trochanteric fractures (HR = 3.2, 95% CI 1.1-9.3).

**Conclusions:**

Impaired functional mobility, physical inactivity, and low body mass may increase the risk for hip fractures with different effects at the cervical and trochanteric levels.

## Background

The incidence of hip fractures is highest worldwide in Scandinavia [1]. In Finland, the number of hip fractures among older people almost doubled between the early eighties and early nineties, but since then, a decline in hip fracture incidence has been observed both in Finland and around the world [1,2]. The total number of hip fractures, however, is likely to rise due to increased longevity [3].

The etiology of hip fractures is multifactorial. The risk factors include female sex and advanced age [4], Caucasian race, [5], low body mass [6], and chronic illnesses [3]. During the last few decades, physical inactivity has been shown to be associated with a greater hip fracture risk among older people [7]. In large, prospective studies, moderate or high leisure time physical activity has been associated with a 28-42% reduction in hip fracture risk [6,8-10]. Physical activity has several advantages, including increased bone strength [11] and decreased risk of falling [12]. During the postmenopausal years, physical activity has been shown to be beneficial for femoral bone density and geometry [11,13,14]. High impact exercise is most beneficial for bone formation and for the maintenance of bone mass [15-17]. Long-term low-impact type of exercise can also decrease bone loss at the proximal femur [14] and can maintain muscle strength and balance, consequently decreasing the risk of falling [18]. Because approximately 90% of all hip fractures occur as the consequences of falls [19], decreasing the risk of falling is also crucial.

Hip fractures can be divided into two principal groups: cervical (femoral neck) fractures and trochanteric fractures. The different etiologies of these fracture types have been observed [20]. In women, bone geometry and pelvic structure have greater effects on cervical fracture risk, while low bone density is associated with trochanteric fractures. Additionally, women with trochanteric fractures appear to be older than women with cervical fractures. Because the risk factors seem to differ between hip fracture types, different strategies might be needed to obtain optimal results in terms of individual fracture risk assessment and hip fracture prevention.

In a previous study, we reported on the different clinical risk factors for cervical and trochanteric fractures [21]. The aim of the present study was to assess further the lifestyle-related risk factors for hip fractures at different bone sites among older female subjects during an extended 13-year follow-up period.

## Methods

### Subjects

**Figure 1:**
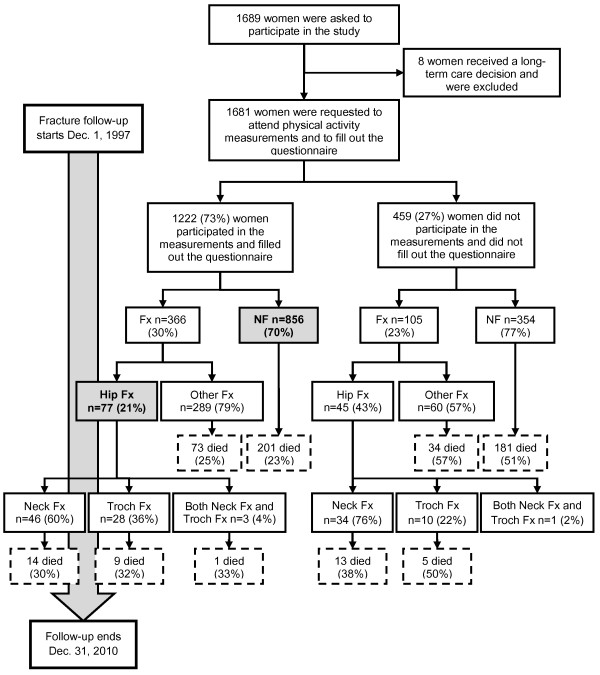
**Participant flow.** Number of women with and without fractures, as well as deaths, in the study population. NF = subjects with no fractures, Fx = subjects with any fracture, Other Fx = subjects with any fracture other than hip fractures, Hip Fx = subjects with at least one hip fracture, Neck Fx = subjects with a cervical fracture, Troch Fx = subjects with a trochanteric fracture. The subjects included to the final analysis have been highlighted.

A detailed description of the study population has been previously published [22]. In brief, all 1681 home-dwelling women, born between 1924 and 1927 residing in Oulu, Finland, in November 1997, were invited to participate in the study (Figure 1). 1222 women (73%) out of them were willing to participate. There were no exclusion criteria. The fracture histories of these 1222 women as well as the 459 non-participants were surveyed for 13 years, from Dec. 1, 1997, to Dec. 31, 2010. The procedures of this study were in accordance with the Declaration of Helsinki. The Ethics Committee of the Northern Ostrobothnia Hospital District approved the study design prior to conducting the study. All of the participants provided written informed consent.

### Methods

The study methods have been described in greater detail elsewhere [22]. Postal questionnaires and interviews were used to collect the baseline data regarding lifestyle factors, including a medical history, smoking habits, alcohol and coffee consumption, lifetime leisure-time physical activity, and calcium intake. The clinical examination included anthropometric measurements and an assessment of functional mobility and dynamic balance using the “Timed Up & Go” (TUG) test [23]. In the TUG test, the subject was timed while she rose from a chair, walked 3 meters, turned, walked back, and sat down again. The TUG test was performed two times consecutively, and the faster time was used in further analyses. In all the tests the subjects were asked to walk at a brisk speed with the help of their standard (if any) walking aid. To assess leisure-time physical activity, a modified Paffenbarger questionnaire was used [24]. The participants were asked to classify themselves according to their frequency and intensity of physical activity during four time periods in their lifespans using a four point scale, corresponding to the ages of 15 years old, 30 years old, and 50 years old and to their current ages. Dairy calcium intake was calculated during the same four time periods of their lifespans [25]. Thirteen-year incident fragility fracture data, regarding hospital-treated fractures in all 1681 women, were collected from hospital discharge registers. A hip fracture was defined as a fracture with ICD-10 (International Classification of Diseases, 10th revision) code between S72.0 and S72.2, S72.0 being classified as a cervical fracture and S72.1 and S72.2 as trochanteric fractures. All of the hip fractures were confirmed manually from medical records to avoid the bias of recording multiple hospitalizations due to a single fracture. The women who only had fractures other than hip fractures were excluded from the final analysis, and subjects with hip fractures were compared to the women with no fractures during the follow up period.

### Statistical analysis

The data were analyzed with PASW Statistics software (Release 18.0.0, IBM Corporation, Route 100, Somers, NY, USA). The participants were classified according to their fracture histories as follows: a) no fracture (NF), b) cervical fracture (Neck Fx), and c) trochanteric fracture (Troch Fx). For the analyses related to leisure exercise exposure, physical activity indices at different ages and a lifetime physical activity index were calculated [22]. The indices were divided into quartiles (very low, low, moderate, and high). Similarly, self-rated mobility was classified into four categories (poor, moderate, good, and very good). Missing calcium intake data for 229 women (18.7%) were replaced with the study population’s mean value. The results of the TUG test were categorized using a cut-off value of 11 seconds, based on a receiver operator characteristics (ROC) analysis. The women with diagnosed heart insufficiency, cerebrovascular disease, or coronary heart disease were aggregated to a cardiovascular disease (CVD) group.

For continuous variables, the differences between the NF and Hip Fx groups were compared using an independent samples *t*-test. For the dichotomous variables, a χ^2^-test was used. Similarly, to compare the NF, Neck Fx, and Troch Fx groups for significant differences, one-way ANOVA (post hoc algorithm: Scheffé’s test) and an independent samples Kruskal-Wallis method were used for continuous and categorical variables, respectively. When comparing the Neck Fx and Troch Fx groups, the first hip fracture type that occurred was selected for analysis in women with multiple hip fractures. Multiple Cox regression analysis was performed to analyze the relative roles of different variables in hip fracture risk. The variables associated with hip fractures in univariate analyses were selected as covariates to calculate hazard ratios (HR) and 95% confidence intervals (95% CI) for different types of hip fractures. In Cox regression analysis, a forward stepwise procedure was used to determine the most predictive variables for hip fracture risk. All regression models were adjusted with age and body mass index (BMI). In stepwise method probability for entry was p < 0.05 and for removal p < 0.10. All women who did not experience a hip fracture during the follow up period (i.e. the women who died during the follow up period or survived to the end of the follow up period without fractures) were considered as censored. For the regression analysis, the two lowest and two highest physical activity categories were combined. In Kaplan-Meier analysis, a log rank test was used to compare the equality of survival distributions for the different physical activity indices (very low or low vs. moderate vs. high). In all of the tests, p-values less than 0.05 were considered statistically significant.

## Results

During the 13-year follow-up period, 366 subjects (30%) out of 1222 sustained a bone fracture (Figure 1). Seventy-seven subjects (6.3%) had a hip fracture. Four women out of 77 had two hip fractures, and one woman had three hip fractures. Forty-nine (4.0%) women had cervical fractures, thirty-one (2.5%) had trochanteric fractures, and three (0.2%) women had both fracture types. In total, 51 of the fractures were cervical, and 32 fractures were located in the trochanteric region. The final study sample (n = 933) consisted of the 77 subjects with hip fracture and the 856 subjects with no fracture.

The mean age of all of the women at the baseline clinical examination was 72 years old. BMI varied between 15 and 46 kg/m^2^ (mean 27 kg/m^2^). The mean weight at baseline was 68 kg (range 40–113 kg), and the mean height was 159 cm (range 139–177 cm). The women with hip fractures were older (p = 0.002), taller (p = 0.012), and thinner (p = 0.041) than the women without fractures (Table 1). During the thirteen-year follow-up, 298 (24.0%) women died (Figure 1). No differences were observed in death rate between the women with and without hip fractures. Additionally, no differences were observed in calcium intake between the participants with hip fractures and the participants without hip fractures. Fewer women with hip fractures had hypertension.

**Table 1:** Baseline characteristics of the women with and without hip fractures (n = 933)

	**NF n = 856**	**Hip Fx n = 77**	**Neck Fx n = 49**	**Troch Fx n = 31**	**p-values**				
					**NF vs. Hip Fx**	**Between groups**	**NF vs. Neck Fx**	**NF vs. Troch Fx**	**Neck Fx vs. Troch Fx**
Age (yrs)	71.3 (1.1)	71.7 (1.1)	71.5 (1.1)	72.1 (0.9)	0.002	< 0.001	0.668	< 0.001	0.031
Age at 1^st^ fracture (yrs)	--	79.4 (3.9)	79.6 (3.8)	79.0 (4.1)	--	0.521	--	--	0.521
Weight (kg)	68.9 (10.9)	66.3 (11.8)	67.2 (12.5)	64.5 (10.2)	0.041	0.076	0.579	0.121	0.611
Height (cm)	157.9 (5.7)	159.6 (5.2)	159.8 (5.1)	159.3 (5.1)	0.012	0.041	0.082	0.438	0.930
BMI (kg/m^2^)	27.7 (4.1)	25.9 (3.8)	26.2 (4.0)	25.3 (3.2)	< 0.001	0.001	0.046	0.008	0.673
Calcium intake / day (mg)	809.1 (345.9)	819.5 (353.9)	857.3 (375.8)	752.7 (294.4)	0.801	0.413	0.618	0.693	0.426
Timed Up & Go (s)	11.1 (3.4)	13.3 (4.9)	12.9 (4.6)	14.4 (5.8)	< 0.001	< 0.001	0.006	< 0.001	0.360
Daily smoking, yes vs. no, n (%)	42 (4.9)	7 (9.1)	4 (8.2)	4 (12.9)	0.115	0.074	0.313	0.049	0.766
	814 (95.1)	70 (90.9)	45 (91.8	27 (87.1)					
Corticosteroids, yes vs. no, n (%)	13 (1.5)	1 (1.3)	0 (0.0)	1 (3.2)	0.879	0.669	0.358	0.454	0.195
	843 (98.5)	76 (98.7)	49 (100.0)	30 (96.8)					
Daily alcohol use, yes vs. no, n (%)	8 (0.9)	1 (1.3)	1 (2.0)	0 (0.0)	0.754	0.798	0.448	0.589	0.434
	848 (99.1)	76 (98.7)	48 (98.0)	31 (100.0)					
Calcium intake <800 mg / day vs. more, n (%)	364 (42.5)	32 (41.6)	18 (36.7)	15 (48.4)	0.870	0.723	0.425	0.517	0.353
	492 (57.5)	45 (58.4)	31 (63.3)	16 (51.6)					
Rheumatoid arthritis, yes vs. no, n (%)	48 (5.6)	7 (9.1)	3 (6.1)	4 (12.9)	0.214	0.273	0.879	0.089	0.265
	808 (49.4)	70 (90.9)	46 (93.9)	27 (87.1)					
Estrogen use, yes vs. no, n (%)	39 (4.6)	5 (6.5)	5 (10.2)	2 (6.5)	0.442	0.084	0.074	0.621	0.072
	817 (95.4)	72 (93.5)	44 (89.8)	29 (93.5)					
Osteoporosis medication, yes vs. no, n (%)	29 (3.4)	3 (3.9)	2 (4.1)	1 (3.2)	0.814	0.973	0.795	0.961	0.875
	827 (96.6)	74 (96.1)	47 (95.9)	30 (96.8)					
Coffee usage > 3 cups / day vs. less, n (%)	384 (44.9)	30 (39.0)	14 (28.6)	16 (51.6)	0.310	0.035	0.019	0.404	0.016
	428 (50.0)	43 (55.8)	33 (67.3)	13 (41.9)					
Diabetes, yes vs. no, n (%)	112 (13.1)	15 (19.5)	9 (18.4)	7 (22.6)	0.117	0.361	0.291	0.128	0.835
	744 (86.9)	62 (80.5)	40 (81.6)	24 (77.4)					
Hypertension, yes vs. no, n (%)	315 (36.8)	19 (24.7)	11 (22.4)	10 (32.3)	0.034	0.043	0.042	0.606	0.314
	541 (63.2)	58 (75.3)	38 (77.6)	21 (67.7)					
CVD, yes vs. no, n (%)	299 (63.2)	26 (33.8)	15 (30.6)	13 (41.9)	0.837	0.483	0.537	0.422	0.272
	557 (34.9)	51 (66.2)	34 (69.4)	18 (58.1)					

Values are the means (SD) unless otherwise specified. Student’s *t*-test (NF vs. Hip Fx) and ANOVA (Scheffé’s post hoc test (NF vs. Neck Fx vs. Troch Fx)) were used with scalar variables, and a χ^2^ test and an independent samples Kruskal-Wallis test with categorical variables were used to calculate differences between groups. NF = subjects with no fractures, Hip Fx = subjects with a hip fracture, Neck Fx = subjects with a cervical fracture, Troch Fx = subjects with a trochanteric fracture, BMI = body mass index, CVD = cardiovascular diseases, SD = standard deviation. Numbers do not match due to missing values.

No differences were observed in lifetime physical activity between the NF and Hip Fx groups nor between the NF, Neck Fx, and Troch Fx groups. However, when comparing physical activity at the age of 72 years (i.e. at baseline), the women with hip fractures were more sedentary than those with no fractures (very low or low vs. moderate or high, p = 0.016). This difference was also observed between the NF and Neck Fx groups (p = 0.003) but not between the NF and Troch Fx groups (p = 0.538). Similarly, high physical activity at the age of 72 protected from cervical hip fractures compared to the more sedentary women (very low, low, or moderate vs. high, p = 0.005 for Neck Fx vs NF, p = 0.02 for Troch Fx). The women with hip fractures had lower functional mobility (p < 0.001). Poor performance in TUG test was associated with an increased risk of both fracture types (p < 0.001). There was no difference in functional mobility between the groups of women with different hip fracture types. Self-rated mobility at baseline was not associated with the risk of hip fractures.

Low functional mobility, measured using the TUG test, was the best independent predictor of both cervical and trochanteric fractures in Cox regression analysis (HR = 3.4, 95% CI 1.8-6.6, and HR = 5.3, 95% CI 2.5-11.4, respectively) (Table 2). Low physical activity in the beginning of the follow-up period was associated with an increased hip fracture risk, especially with the risk of cervical fractures (HR = 2.5, 95% CI 1.3-4.9). There was no relationship between fractures and physical activity earlier in life. Moderate coffee consumption (>3 cups per day) and arterial hypertension decreased the risk of cervical fractures (HR = 0.4, 95% CI 0.2-0.8, and HR = 0.4, 95% CI 0.2-0.8, respectively), whereas daily smoking was a risk factor for trochanteric fractures (HR = 3.2, 95% CI 1.1-9.3). Low BMI was associated with both fracture types, as well as with a general hip fracture risk (HRs between 0.83 and 0.89). Neither estrogen treatment nor specific osteoporosis medication at baseline was associated with the future risk of hip fractures.

**Table 2:** Cox regression models for having any hip fractures, cervical fractures, and trochanteric fractures in a population-based sample of older women

	**β**	**SE**	**HR**	**95% CI for HR**	**Covariate p-value**
*Any hip fractures*^*a)*^					
Age / 1 year increment	0.257	0.113	1.29	1.04 - 1.61	0.022
BMI / 1 unit increment	−0.144	0.034	0.87	0.81 - 0.93	< 0.001
TUG ≥ 11 s vs. less (referent)	1.201	0.258	3.32	2.01 - 5.51	< 0.001
Low physical activity vs. moderate to high (referent)	0.697	0.259	2.01	1.21 - 3.34	0.007
Hypertension vs. none (referent)	−0.627	0.306	0.53	0.29 - 0.97	0.040
*Cervical fractures*^*b)*^					
BMI / 1 unit increment	−0.119	0.042	0.89	0.82 - 0.96	0.004
TUG ≥ 11 s vs. less (referent)	1.226	0.333	3.41	1.77 - 6.55	< 0.001
Low physical activity vs. moderate to high (referent)	0.910	0.344	2.48	1.27 - 4.87	0.008
Hypertension vs. none (referent)	−1.030	0.426	0.36	0.16 - 0.82	0.016
Coffee consumption > 3 cups / day vs. less (referent)	−0.944	0.358	0.39	0.19 - 0.79	0.008
*Trochanteric fractures*^*c)*^					
Age / 1 year increment	0.636	0.183	1.89	1.32 - 2.70	0.001
BMI / 1 unit increment	−0.186	0.051	0.83	0.75 - 0.92	< 0.001
TUG ≥ 11 s vs. less (referent)	1.664	0.393	5.28	2.45 - 11.41	< 0.001
Daily smoking vs. no (referent)	1.165	0.543	3.21	1.11 - 9.29	0.032

Hazard ratios (HR) are calculated compared to the no fracture group. In the cervical fracture model, age was also added as a covariate but was not included in the final model. β = regression coefficient, SE = standard error of β, CI = confidence interval, BMI = body mass index, TUG = “Timed Up & Go” test. Due to missing values, the number of subjects were ^a)^ Fx n = 66, NF n = 775, ^b)^ Fx n = 39, NF n = 740, and ^c)^ Fx n = 31, NF n = 856.

**Figure 2:**
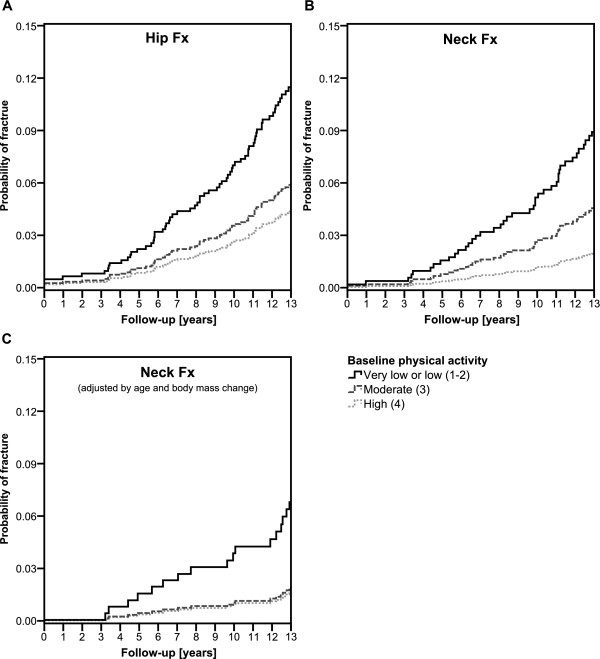
Probability of (A) any hip fracture and (B) cervical fracture with different baseline physical activity indices (very low or low, moderate, and high) adjusted by age and body mass index at baseline, and of (C) cervical fracture after adjustment for age and change in body weight between baseline and 2004 (NF n = 488, Neck Fx n = 17).

A significant difference between the baseline physical activity categories (very low or low vs. moderate vs. high) was found in Kaplan-Meier survival analysis, with the exception of trochanteric fractures (Figure 2). High physical activity protected the women from hip fractures (p-values of log rank tests: Hip Fx p = 0.012; Neck Fx p = 0.002; Troch Fx p = 0.578). After adjusting the model for the change in BMI between 1997 and 2004, the protective effect of physical activity against cervical hip fractures, but not all hip fractures, remained (p = 0.017).

## Discussion

In this prospective, population-based, 13-year cohort study, low physical activity was a strong risk factor for cervical, but not for trochanteric, hip fractures. Additionally, functional mobility, measured with the TUG test, and low BMI were associated with the risk of both hip fracture types. Other clinical factors appeared to differ between the hip fracture sites: hypertension and coffee consumption of more than three cups per day decreased the risk of cervical hip fractures, while daily smoking increased the risk of trochanteric hip fractures.

Physical activity helps to maintain muscle strength and mobility and thus prevent falls in older people [18]. In contrast, a more active lifestyle has been shown to increase the incidence of nonsyncope falls and related traumas, e.g., wrist fractures [14,26]. Our results showed that the women with high physical activity at baseline had a lower risk of future hip fractures (especially the risk of cervical fractures) than women with moderate or low activity. A similar dose–response relationship has been reported in both male [27] and female populations [6,28]. Based on our results, regardless of earlier lifetime physical activity, an active lifestyle in the postmenopausal years decreases the fracture risk. Both Feskanich et al. [28] and Michaëlsson et al. [27] reported that by increasing physical activity during the lifespan, the fracture risk could be diminished, whereas Høidrup et al*.*[10] reported that increments in physical activity during follow-up did not influence the risk of hip fractures. In our study, no longitudinal data on physical activity during the follow-up period were available.

Baseline physical activity was associated with cervical, but not with trochanteric, fractures in our study population. This finding might indicate an association between physical activity and femoral geometry. Previously, it has been shown that bone geometry is associated with cervical fracture risk, while bone density is more strongly related to the risk of trochanteric fractures [29]. Furthermore, it has been shown that mechanical loading is a strong external determinant of the structure and concomitant strength of the femoral neck [30]. Therefore, this finding may arise from the structural weakening of the femoral neck caused by a low physical activity level. In particular, the thinning with age of the superolateral femoral neck cortex leads to the loss of elastic stability due to under loading of this site, exposing it to local buckling of the cortex [31,32]. Based on the current results, it can be assumed that this structural weakening may accelerate in individuals with low levels of physical activity, thus increasing the risk of femoral neck failure.

In this study, we evaluated functional mobility using the “Timed Up & Go” (TUG) test. The TUG test is easy to perform and reproducible, and it is a sensitive and specific measure for evaluating fall risks [23,33]. In our study, 11 seconds was selected as the threshold value for the TUG test based on receiver operator characteristics (ROC) analysis. Thirty-three percent of the subjects took more than eleven seconds to complete the test. We found low functional mobility to be a risk factor for both types of hip fractures. Similar results with a similar population were reported earlier for general hip fracture risks among subjects with slow TUG performances [34]. In some studies, walking speed and repeated rising from a chair have been used as alternatives to the TUG test to assess mobility and neuromuscular function. In a study by Fox et al. [35], walking speed was associated with both hip fracture types, but the ability to complete five chair stands was not associated with either. In contrast, Cummings et al. [8] reported that subjects unable to rise repeatedly from a chair had a higher risk of hip fractures. Because functional mobility is easy to assess, it should be routinely evaluated to screen for individuals at high risk for fragility hip fractures.

The role of arterial hypertension as a risk factor for hip fractures is somewhat controversial. Hypertension alone has been shown to increase fracture risks by affecting calcium metabolism [36], as well as by increasing the risk of falling due to reduced baroreflex sensitivity or hypotension [37]. In the present study, hypertension was found to be protective against hip fractures. This may be due to the use of thiazides as diuretic medication for hypertension. Thiazide diuretics have been reported to have positive effects, in terms of bone strength [38], by reducing urinary calcium excretion and helping to maintain calcium balance [39], and by inhibiting bone resorption by means of inducing metabolic alkalosis [40].

Multiple studies have indicated that smoking increases fracture risks in both women and men [41,42]. Numerous reasons for increased fracture risks have been suggested. These reasons include direct toxic effects on the bones due to exposure to nicotine, reduction of calcium absorption, transient increases in cortisol levels after smoking, lower BMI, and an increased risk of falling in smokers, as well as lower estrogen levels and earlier menopause [43]. In our population, there were only seven fracture subjects who declared at baseline that they smoked daily. The observed increase in trochanteric fracture risk may be due to the above-mentioned reasons, but the ultimate reason cannot be determined. Because the trochanteric region is rich in trabecular bone, the increased risk might occur because tobacco affects this metabolically active region. However, the small number of fractures limits the statistical power of this finding. Nevertheless, our results suggest an increased risk of trochanteric fractures among smokers and are in line with earlier studies.

Our results suggest that coffee consumption of more than 3 cups per day may prevent cervical hip fractures. However, excessive coffee drinking has been reported to be associated with an increased hip fracture risk [8,21]. In a recent review by Higdon and Frei [44], the effects of coffee on bone density and hip fracture risk were discussed. Caffeine affects calcium absorption and leads to a slightly negative calcium balance in individuals with inadequate calcium intake. Moderate coffee consumption has some other health benefits [44], which together with our finding support moderate coffee consumption.

We found no differences in hip fracture risks between women who had received estrogen treatment or taken osteoporosis medication at baseline compared to women who did not receive estrogen treatment. One possible explanation for this result is that the new-generation drugs for osteoporosis were not available earlier in the study period, and very few women had taken medication for osteoporosis. Furthermore, population studies have observed a reduced risk of hip fractures with postmenopausal hormone use among sedentary women but not among physically active women [10,27]. However, we did not observe this type of trend. This finding might be due to the low number of fractures, especially among women taking estrogen medication. The postal questionnaires also included questions concerning asthma and incontinence. These conditions, however, showed no effects on the hip fracture risk in this population.

The strengths of this study were its population-based, prospective nature and the long follow-up period. The target population was a homogenous, stable, and representative sample of older Finnish women, obtained from the National Population Register of Finland, which provides 100% coverage. This study also has some limitations. Because of the population-based nature of the present study, the results can be generalized to older Caucasian women. However, there might be some selection bias because the 459 women (27.3% of the total cohort of 1681 women ) who neither replied to the postal questionnaires nor participated in the clinical examination were more fragile, with a higher hip fracture rate (9.8% vs. 6.3%) and higher mortality (50.8% vs. 25.1%) than the participants. According to Finnish National Institute for Health and Welfare, in year 2009, 6085 hip fractures occurred in Finland. The age-standardized hip fracture incidence of 293 fractures /100,000 persons among Finnish women has recently been reported [45]. Thus, the results may not be suitable for generalization to very frail or institutionalized women. Unfortunately, other health data were not available here for the non-participants. The number of hip fractures was limited. In 1222 women, we observed 49 cervical and 31 trochanteric fractures. It also should be noted that all of the clinical and questionnaire data, excluding the fracture data, were collected only at baseline and were not repeatedly collected during the follow-up. In addition, no hip or spine DXA data were available.

## Conclusions

In conclusion, the differences between cervical and trochanteric fracture risk factors were observed. Low baseline physical activity and impaired functional mobility, along with low BMI, increased the risk, whereas moderate coffee consumption and arterial hypertension decreased the risk of cervical fractures. Impaired functional mobility, smoking, advanced age, and low BMI predicted trochanteric hip fractures. Further studies on the effect of impaired mobility and low physical activity are needed to confirm their independent role in hip fracture risk stratification.

## Abbreviations

HR: Hazard Ratio; CI: Confidence Interval; TUG: Timed Up & Go -test; ICD-10: International Classification of Diseases 10th revision; ROC: Receiver Operator Characteristics; CVD: Cardiovascular disease; ANOVA: Analysis of variance; BMI: Body Mass Index; β: Regression coefficient; SE: Standard Error of regression coefficient.

## Competing interests

The authors declare that they have no competing interests.

## Authors’ contributions

MM participated in the fracture data collection, carried out the main analyses, and wrote the main parts of the manuscript. ET and HJ participated in the fracture data collection and analyses of the data. PP made a contribution to the design of the study and revising the manuscript JK, JH, and SK-K participated in conception and design of the study and JH also participated in data collection. TJ and RK participated in conception and design and made substantial contributions to the manuscript. RK also participated significantly to data collection. All authors have read, revised, and given their final approval of the version to be published.

## Pre-publication history

The pre-publication history for this paper can be accessed here:

http://www.biomedcentral.com/1471-2474/13/173/prepub
